# Real-world comparison of mono and dual combination therapies of metformin, sulfonylurea, and dipeptidyl peptidase-4 inhibitors using a common data model: A retrospective observational study

**DOI:** 10.1097/MD.0000000000028823

**Published:** 2022-02-25

**Authors:** Kyung Ae Lee, Heung Yong Jin, Yu Ji Kim, Sang Soo Kim, Eun-Hee Cho, Tae Sun Park

**Affiliations:** a Division of Endocrinology and Metabolism, Department of Internal Medicine, Research Institute of Clinical Medicine of Jeonbuk National University-Biomedical Research Institute of Jeonbuk National University Hospital, Jeonbuk National University Medical School, Jeonju, Korea; b Division of Endocrinology and Metabolism, Department of Internal Medicine, Biomedical Research Institute, Pusan National University Hospital, Busan, Korea; c Department of Internal Medicine, School of Medicine, Kangwon National University, Chuncheon, Korea.

**Keywords:** diabetic complications, glycemic control, hypoglycemic agent, type 2 diabetes mellitus

## Abstract

The comparative effectiveness of oral hypoglycemic agents on glycemic control and chronic complications in clinical practice is unknown in Korea. This study aimed to compare glycemic control and the incidence of hypoglycemia and chronic complications among adult patients with type 2 diabetes prescribed metformin, dipeptidyl peptidase-4 inhibitors (DPP4I), and sulfonylurea (SU) as monotherapy or dual combination therapy.

We retrospectively analyzed propensity-matched cohort data from 3 national university hospitals in Korea. All electronic health records were transformed into a unified Observational Medical Outcomes Partnership Common Data Model and analyzed using ATLAS, an open-source analytical tool, and R software. Glycemic control was assessed as the first observation of a reduction in glycosylated hemoglobin (HbA1c) level below 7% after prescription of the drug. Differences in the incidence of chronic complications were compared based on the first observation of each complication. Glycemic control and chronic complications were evaluated in patients who maintained the same prescription for at least 3 and 12 months, respectively.

Patients who received metformin had lower hazard of reaching HbA1c levels below 7% as compared with those who received SU, and had higher hazard compared with those who received DPP4I (hazard ratio [HR], 0.86; 95% confidence interval [CI], 0.75–0.98; and HR, 1.68; 95% CI, 1.42–1.99, respectively). The incidence of hypoglycemia was significantly higher in the SU group than in the metformin and DPP4I groups (metformin vs SU; HR, 0.30; 95% CI, 0.21–0.43; SU vs DPP4I; HR, 4.42; 95% CI, 2.35–8.31). Metformin + DPP4I had similar hazard of reaching HbA1c levels below 7% compared with metformin + SU (HR, 1.19; 95% CI, 0.99–1.43) and the incidence of hypoglycemia was significantly lower in the metformin + DPP4I group (HR 0.13; 95% CI 0.05–0.30). There was no significant difference in the analysis of the occurrence of chronic complications.

SU followed by metformin was effective, and both drugs showed an increased hazard of reaching HbA1c levels below 7% compared with DPP4I. Metformin + DPP4I is comparatively effective for HbA1c level reduction below 7% compared with metformin + SU. Hypoglycemia was high in the SU-containing therapy.

## 1 Introduction

The global incidence of diabetes mellitus (DM) is increasing, and type 2 diabetes mellitus (T2D) accounts for most cases.^[1]^ Acute and chronic complications of DM are major public health burdens; therefore, efforts to reduce them are important. Guidelines recommend metformin as initial monotherapy for T2D if there are no contraindications, based on its perceived benefits pertaining to weight gain, tolerability, and cost.^[2,3]^ Combination therapy with other drugs is recommended when the blood glucose level is high following monotherapy or at the time of initial diagnosis.^[2,3]^ Anti-diabetic drugs (ADDs) with varying mechanisms have been developed, taking into account the complex pathophysiology of T2D.^[4]^ Six classes of oral hypoglycemic agents (OHAs) are available in South Korea. Among these, metformin, dipeptidyl peptidase-4 inhibitors (DPP4I), and sulfonylurea (SU) are the most commonly prescribed OHAs for T2D.^[5]^ The efficacy and safety of each drug have been validated in prospective randomized clinical trials (RCTs) and cohort studies.^[6]^ In addition to its glucose-lowering efficacy, the influence of ADD on chronic complications, particularly cardiovascular and renal complications, is known.^[2,7]^ However, the comparative effectiveness of each drug on glycemic control and chronic complications in real clinical practice, such as monotherapy or dual combination therapy, is unknown, especially in Korean patients. A real-world study with large-scale data can address this question.

The Observational Health Data Sciences and Informatics (OHDSI) is a global consortium founded in 2008 to support data-based medical research. The OHDSI has a research network with federated data harmonized with the Observational Medical Outcomes Partnership Common Data Model (OMOP-CDM), a unified database model that integrates real-world data (RWD) sources, including electronic health records (EHRs) with the same standards.^[8]^ The CDM facilitates multicenter analysis, and CDM-based RWD is useful for elucidating various treatment pathways or sequences and assessing differences in outcomes.^[9]^ This has been used in a multinational study evaluating the association of glycosylated hemoglobin (HbA1c) with the use of OHAs in patients with T2D treated with metformin.^[10]^ Therefore, the current study aimed to compare glycemic control, the incidence of hypoglycemia, and chronic complications among patients with T2D treated with frequently prescribed OHAs, using OMOP-CDM.

## 2 Methods

### 2.1 Study design and data sources

The present was a retrospective, observational, multicenter study that employed EHRs, which were transformed into a unified OMOP-CDM. Data from 3 national university hospitals in Korea were used: Jeonbuk National University Hospital from 1988 to 2020, Kangwon National University Hospital from 2003 to 2020, and Pusan National University Hospital from 2011 to 2019. The requirement for informed consent for this study was waived by the institutional review board (IRB), as patient privacy was maintained by anonymizing data sources and distributed data models.

### 2.2 Patient selection

We used a specific combination of ADDs, diagnosis codes, and laboratory values to identify patients with T2D who received OHAs. The cohort included patients aged 18 years or older with T2D. T2D was defined as having at least 1 of the following criteria: the International Classification of Disease 10th revision (ICD-10) diagnostic codes (E11–14) as a principal or additional diagnosis, HbA1c ≥6.5%, and fasting serum glucose ≥126 mg/dL. Patients with type 1 (ICD-10 diagnostic codes of E10) or gestational DM (ICD-10 diagnostic code O24) were excluded from the study. Within this T2D cohort, patients prescribed OHA monotherapy (metformin, DPP4I including 9 types of gliptins, and SU including glimepiride and gliclazide) and combination therapy (metformin + SU, metformin + DPP4I, and SU + DPP4I) were selected. In the case of a patient receiving monotherapies or dual combination therapies at different times during the study period, the patient was included in both groups. Subjects who were prescribed an ADD other than the drug to be analyzed within the analysis period were excluded. All ADDs were categorized according to their mechanism of action rather than their ingredients. Considering the need for a reasonable exposure time, the glycemic control analysis included patients who maintained the same prescription for at least 3 months and had at least 1 HbA1c test after drug prescription. Chronic complication analysis included patients who maintained the same prescription for at least 12 months. Hypoglycemic analysis was performed when the drug was maintained for at least 3 months.

### 2.3 Outcomes

Glycemic control was assessed as the first observation of a reduction in HbA1c levels below 7% following drug administration. Hypoglycemia was categorized as either severe or non-severe. Severe hypoglycemia was defined as a diagnostic code of hypoglycemia (ICD-10 code E16.2) or a case in which 50% glucose fluid was prescribed in an emergency room visit. Non-severe hypoglycemia was defined as a serum or glucometer-measured glucose level of less than 70 mg/dL. The difference in the incidence of chronic complications was compared based on the first observation of each complication following drug prescription. The chronic complications considered were ischemic heart disease (IHD), heart failure (HF), and ischemic stroke, defined by their ICD-10 codes (I20-25 for IHD; I11, I13, and I50 for HF; I63-64, I693-694, and G45 for ischemic stroke). Diabetic retinopathy was defined as ICD-10 code (H36, E1120-22, E1220-22, E1320-22, and E1420-22) or the prescription of a retinopathy drug (calcium dobesilate). Diabetic neuropathy was defined as the prescription of neuropathy drugs (pregabalin, duloxetine, tricyclic antidepressants, and alpha-lipoic acid). Diabetic nephropathy was defined as 1 or more of the following: diagnostic codes for nephropathy (ICD-10 code N18) or albuminuria of 30 mg/g or more, and a glomerular filtration rate of less than 60 mL/min/1.73 m^2^. In all analyses for chronic complications, patients with a pre-existing event prior to taking the drug were excluded from the analysis.

### 2.4 Statistical analysis

All analyses in this study were performed using ATLAS, an open-source analytical tool developed by the OHDSI community^[11]^ and R (version 4.0.5). ATLAS is a publicly available web-based tool that facilitates the design and execution of analyses on standardized, patient-level, observational data in the CDM format.^[8,12]^ We performed 3 pairwise comparisons in monotherapy (metformin vs SU, metformin vs DPP4I, and SU vs DPP4I) and dual combination therapy (metformin + DPP4I vs metformin + SU, metformin + DPP4I vs SU + DPP4I, and metformin + SU vs SU + DPP4I). Outcomes were assessed using hazard ratios (HRs) and estimated using a Cox proportional hazards model with a 95% confidence interval (CI). Patients were censored at the time of other ADDs prescription and at their last recorded time of follow-up. If the outcome event occurred more than once, only the first occurrence was considered. Propensity score matching (PSM) was performed to adjust for confounding variables. Covariates including age and sex (for all analyses), baseline HbA1c (for hypoglycemia and chronic complication analysis), and statin use (chronic complication analysis only) were used in PSM. The HRs of each outcome from each study site were analyzed after matching the covariates sequentially. Subsequently, the overall results were evaluated using the meta-analysis method, a statistical analysis combining the results of 3 hospitals using the meta package in R, version 4.19.0. If the heterogeneity *P* value was < 0.05, the random-effects model was used; otherwise, the fixed-effects model was applied. For the analysis of hypoglycemia and chronic complications, data from only those institutions that could be analyzed were used.

### 2.5 Ethics statement

Each site obtained IRB approval for the analysis (IRB number 2019-11-056 in Jeonbuk National University Hospital). The present study was conducted in accordance with the Code of Ethics of the World Medical Association (Declaration of Helsinki) involving humans.

## 3 Results

### 3.1 Study population

**Figure 1:**
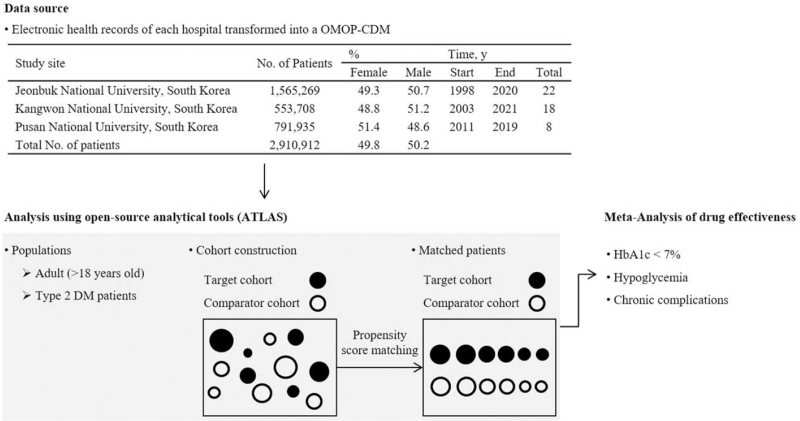
The flowchart of study participants in the common data model network. Patient data at the 3 study sites were transformed into OMOP-CDM. All analyses were performed using ATLAS, an open-source analytical tool developed by the OHDSI community, and R (version 4.0.5). DM = diabetes mellitus, HbA1c = glycated hemoglobin, OHDSI = Observational Health Data Sciences and Informatics, OMOP-CDM = Observational Medical Outcomes Partnership Common Data Model.

Figure 1 illustrates the workflow of this study. Data from 2,910,912 patients (1,462,129 men, 50.2%) were included in the 3 data sources. Table 1 shows the total number of patients in the cohort used for the HbA1c outcome analysis for each pairwise comparison and in each data source, before and after PSM in monotherapy and dual combination therapy, respectively. The number of patients before and after matching for each drug comparison for hypoglycemia and chronic complications is provided in Table S1, Supplemental Digital Content. In all 3 institutions, metformin was the most common monotherapy, and metformin + DPP4I was the most common dual combination therapy in the treatment of T2D.

**Table 1:** Number of patients before and after matching for each drug comparison and HbA1c outcome.

	JNUH				KNUH				PNUH			
	Unmatched		Matched^∗^		Unmatched		Matched^∗^		Unmatched		Matched^∗^	
Oral hypoglycemic agents	T	C	T	C	T	C	T	C	T	C	T	C
Metformin (T) vs SU (C)	1542	1147	1147	1147	1026	556	556	556	1622	243	243	243
Metformin (T) vs DPP4I (C)	1542	745	721	721	1147	745	702	702	1026	489	484	484
SU (T) vs DPP4I (C)	1147	745	702	702	556	489	461	461	243	293	226	226
Metformin + DPP4I (T) vs Metformin + SU (C)	402	247	241	241	331	169	169	169	597	328	328	328
Metformin + DPP4I (T) vs SU + DPP4I (C)	402	60	60	60	331	35	35	35	597	49	49	49
Metformin + SU (T) vs SU + DPP4I (C)	247	60	56	56	169	35	35	35	328	49	49	49

C = comparator, DPP4I = dipeptidyl peptidase-4 inhibitors, HbA1c = glycosylated hemoglobin, JNUH = Jeonbuk National University Hospital, KNUH = Kangwon National University Hospital, PNUH = Pusan National University Hospital, SU = sulfonylurea, T = target.

∗ Propensity score matching covariates: sex, age.

### 3.2 Comparison of metformin, SU, and DPP4I monotherapy

#### 3.2.1 Glycemic control and hypoglycemia

**Figure 2:**
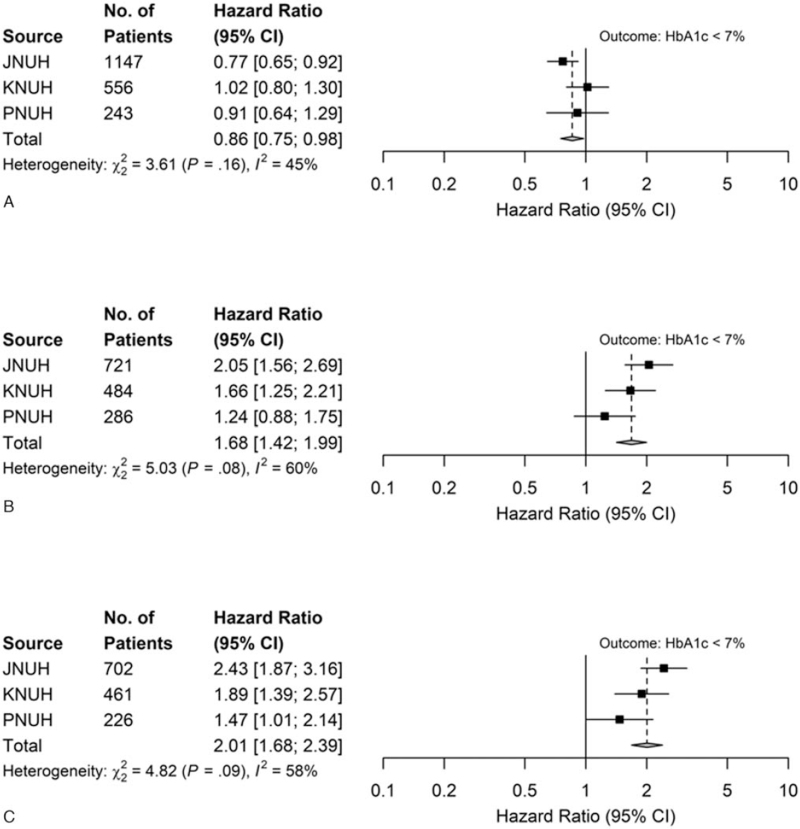
Hazard ratios (HRs) for the comparative effectiveness of monotherapy (after meta-analysis). (A) Outcomes: HbA1c levels <7%: metformin (T) vs SU (C). (B) Outcomes: HbA1c levels <7%: metformin (T) vs DPP4I (C). (C) Outcomes: HbA1c levels <7%: SU (T) vs DPP4I (C). A hazard ratio greater than 1 implies target drug is associated with a higher hazard of reaching HbA1c < 7% compared with comparator drug. C = comparator, CI = confidence interval, DPP4I = dipeptidyl peptidase-4 inhibitors, HbA1c = glycosylated hemoglobin, JNUH = Jeonbuk National University Hospital, KNUH = Kangwon National University Hospital, PNUH = Pusan National University Hospital, SU = sulfonylurea, T = target.

The number of patients, HRs for each drug comparison, and outcomes across all 3 hospitals are shown in Table S2, Supplemental Digital Content. Figure 2 presents the HRs of each OHA for HbA1c levels <7% obtained from the meta-analysis. Metformin monotherapy had lower hazard of reaching HbA1c levels below 7% compared to SU treatment and had higher hazard of reaching HbA1c levels below 7% compared to DPP4I (HR, 0.86; 95% CI, 0.75–0.98; and HR, 1.68; 95% CI, 1.42–1.99, respectively; Fig. 2A, B). The hazard of a reduction in HbA1c levels below 7% in the SU group was higher than that in the DPP4I group (HR, 2.01; 95% CI, 1.682–2.39; Fig. 2C). The incidence of hypoglycemia was significantly higher in the SU group than in the metformin and DPP4I monotherapy groups (metformin vs SU; HR, 0.30; 95% CI, 0.21–0.43; SU vs DPP4I; HR, 4.42; 95% CI, 2.35–8.31); there was no significant difference between the metformin and DPP4I groups (HR, 1.04; 95% CI 0.59–1.84) (Table 2).

**Table 2:** Hazard ratios (HRs) for hypoglycemia and chronic complications of monotherapy (after meta-analysis).

	Consensus hazard ratio (95% CI)		
Outcome	Metformin (T) vs SU (C)	Metformin (T) vs DPP4I (C)	SU (T) vs DPP4I (C)
Hypoglycemia^∗^	0.33 (0.23–0.46)^§^	1.02 (0.56–1.86)^§^	3.32 (2.05–5.39)
Hypoglycemia^†^	0.30 (0.21–0.43)	1.04 (0.59–1.84)	4.42 (2.35–8.31)^||^
IHD^∗^	0.64 (0.43–0.97)	0.92 (0.34–2.45)	1.11 (0.35–3.57)
IHD^†^	0.63 (0.42–0.94)	0.92 (0.54–1.54)	1.00 (0.60–1.68)
IHD^‡^	0.65 (0.24–1.76)	0.79 (0.46–1.38)	1.30 (0.43–3.92)
Heart failure^∗^	0.83 (0.50–1.38)	0.87 (0.20–3.66)	1.22 (0.71–2.08)
Heart failure^†^	0.85 (0.52–1.38)	0.85 (0.17–4.28)	1.45 (0.81–2.61)
Heart failure^‡^	0.61 (0.36–1.03)	0.84 (0.45–1.56)	1.08 (0.60–1.96)
Ischemic stroke^∗^	0.65 (0.39–1.08)	1.03 (0.48–2.24)	1.71 (0.83–3.50)
Ischemic stroke^†^	0.65 (0.39–1.07)	1.18 (0.58–2.43)	1.80 (0.85–3.81)
Ischemic stroke^‡^	0.71 (0.43–1.19)	1.17 (0.54–2.49)	1.87 (0.85–4.11)
Diabetic retinopathy^∗^	1.13 (0.81–1.56)	1.03 (0.63–1.52)	0.95 (0.64–1.41)
Diabetic retionopathy^†^	1.03 (0.74–1.43)	0.94 (0.63–1.41)	0.78 (0.49–1.25)
Diabetic retinopathy^‡^	1.30 (0.93–1.82)	0.84 (0.56–1.27)	0.94 (0.58–1.53)
Diabetic neuropathy^∗^	0.86 (0.67–1.11)	1.17 (0.79–1.74)	1.32 (0.89–1.95)
Diabetic neuropathy^†^	0.75 (0.58–0.97)	1.19 (0.80–1.77)	1.43 (0.95–2.15)
Diabetic neuropathy^‡^	0.79 (0.60–1.04)	1.21 (0.80–1.84)	1.26 (0.83–1.91)
Diabetic nephropathy^∗^	0.52 (0.20–1.38)	1.01 (0.69–1.46)	1.15 (0.79–1.66)
Diabetic nephropathy^†^	0.67 (0.47–0.96)	0.68 (0.45–1.01)	1.22 (0.82–1.82)
Diabetic nephropathy^‡^	0.72 (0.51–1.02)	0.81 (0.55–1.20)	1.10 (0.71–1.70)
UACR ≥ 30^∗^	0.66 (0.21–2.06)	1.03 (0.64–1.65)	1.29 (0.78–2.15)
UACR ≥ 30^†^	0.60 (0.18–2.04)	0.88 (0.35–2.17)	1.27 (0.74–2.20)
UACR ≥ 30^‡^	0.69 (0.24–2.01)	0.91 (0.39–2.17)	0.95 (0.54–1.69)

C = comparator, CI = confidence interval, DPP4I = dipeptidyl peptidase-4 inhibitors, HbA1c = glycated hemoglobin, IHD = ischemic heart disease, JNUH = Jeonbuk National University Hospital, KNUH = Kangwon National University Hospital, PNUH = Pusan National University Hospital, SU = sulfonylurea, T = target, UACR = urine albumin-creatinine ratio.

∗ Propensity score matching covariates: sex, age.

† Propensity score matching covariates: sex, age, HbA1c.

‡ Propensity score matching covariates: sex, age, HbA1c, statin.

§ Meta-analysis result of JNUH and KNUH because data from PNUH were unavailable.

|| Analysis result of JNUH because data from KNUH and PNUH were unavailable.

#### 3.2.2 Chronic complications

The results of the chronic complications are summarized in Table 2. The metformin group had a significantly lower incidence of IHD (HR 0.63; 95% CI 0.42–0.94), diabetic neuropathy (HR 0.75; 95% CI 0.58–0.97), and diabetic nephropathy (HR 0.67; 95% CI 0.47–0.96), after PSM for age, sex, and baseline HbA1c levels, compared to the SU group. However, these differences disappeared when PSM included an additional variable, the use of statins. The occurrence of HF was lower in the metformin group than in the SU group; however, this difference was not statistically significant (HR, 0.61; 95% CI 0.36–1.03). There was no significant difference in the occurrence of chronic complications after PSM between the metformin and DPP4I groups and between the SU and DPP4I groups.

### 3.3 Dual combination therapies of metformin, SU, and DPP4I

#### 3.3.1 Glycemic control and hypoglycemia

**Figure 3:**
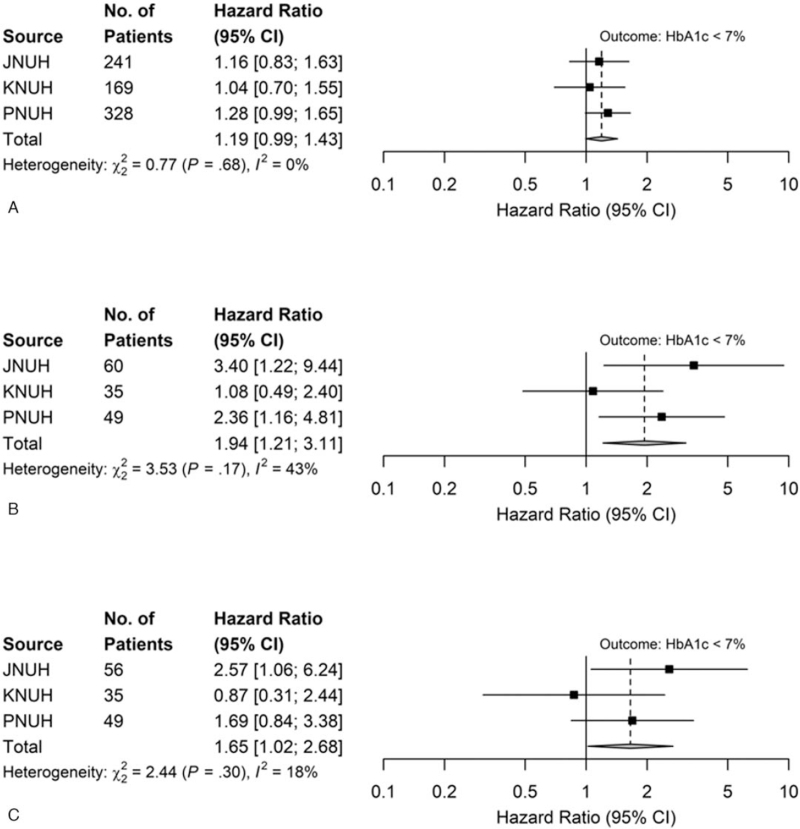
Hazard ratios (HRs) for the comparative effectiveness of dual therapy (after meta-analysis). (A) Outcomes: HbA1c levels <7%: metformin + DPP4I (T) vs metformin + SU (C). (B) Outcomes: HbA1c levels <7%: metformin + DPP4I (T) vs SU + DPP4I (C). (C) Outcomes: HbA1c levels <7%: metformin + SU (T) vs SU + DPP4I (C). A hazard ratio greater than 1 implies target drug is associated with a higher hazard of reaching HbA1c < 7% compared with comparator drug. CI = confidence interval, DPP4I = dipeptidyl peptidase-4 inhibitors, HbA1c = glycated hemoglobin, JNUH = Jeonbuk National University Hospital, KNUH = Kangwon National University Hospital, PNUH = Pusan National University Hospital, SU = sulfonylurea.

When the metformin-based dual combinations were compared, metformin + DPP4I showed a slightly higher hazard of reaching HbA1c levels below 7% than metformin + SU; however, this was not statistically significant (HR, 1.19; 95% CI, 0.99–1.43, Fig. 3A). Metformin + DPP4I showed higher hazard of reduction in HbA1c levels below 7% than SU + DPP4I (HR, 1.94; 95% CI 1.21–3.11, Fig. 3B). Metformin + SU presented a significantly higher hazard of reaching HbA1c levels below 7% than SU + DPP4I (HR, 1.65; 95% CI 1.02–2.68, Fig. 3C). The incidence of hypoglycemia was significantly lower in the metformin + DPP4I group than in the metformin + SU group (HR 0.13; 95% CI 0.05–0.30). There were no significant differences among the other groups (Table S3, Supplemental Digital Content).

#### 3.3.2 Chronic complications

The chronic complications are summarized in Table S3, Supplemental Digital Content. The analysis was limited because of insufficient data that fit the inclusion or exclusion criteria for chronic complication analysis in each hospital. The metformin + DPP4I group had a significantly lower incidence of diabetic nephropathy (HR, 0.65; 95% CI 0.45–0.93) than the metformin + SU group after PSM for age and sex. However, these differences disappeared when the PSM included additional variables, HbA1c levels, and the use of statins. No significant differences were observed between the groups.

## 4 Discussion

Recently, various ADDs with different mechanisms have been used for glycemic control in the treatment of T2D. T2D is a heterogeneous disorder resulting from complex pathophysiology.^[13]^ Therefore, it is important to evaluate the effectiveness of each ADD in practice. This study evaluated the effectiveness of frequently prescribed OHAs as monotherapy and dual combination therapy using large-scale RWD in Korean patients. Monotherapy with metformin or SU was comparatively effective; SU was slightly more effective than metformin. Treatment with both drugs showed an increased hazard of reduction in HbA1c levels below 7% than treatment with DPP4I alone. Hypoglycemia was significantly higher in the SU group. The occurrence of chronic complications was similar across the drug monotherapies after PSM for covariables. In dual combination therapy, metformin + DPP4I was comparatively effective for HbA1c level reduction below 7% compared with metformin + SU. Both metformin + DPP4I and metformin + SU were more effective for HbA1c reduction than SU + DPP4I. Hypoglycemia was high in the SU-containing group, and chronic complications were not significantly different among the 3 groups.

Metformin is effective, safe, inexpensive, and reduces the risk of cardiovascular events and death.^[14]^ Therefore, most clinical practice guidelines for T2D suggest metformin as first-line therapy. However, if patients have contraindications or are intolerant to metformin, initial therapy is chosen based on patient factors, considering drugs from other classes.^[2,3]^ Along with metformin, SU has been used for a long time in clinical practice; therefore, there are many comparative studies on their effectiveness as first-line drugs. However, few studies have been conducted on recently developed OHAs. Compared with SU in a previous meta-analysis, metformin as a first-line monotherapy was beneficial in terms of HbA1c levels, weight gain, and cardiovascular mortality.^[15]^ On the other hand, patients with uncontrolled hyperglycemia associated with T2D can be effectively treated with SU.^[16]^ Another meta-analysis revealed that second- and third-generation SUs do not influence all-cause or cardiovascular mortality, but they decrease the risk of non-fatal macrovascular outcomes compared with metformin.^[17]^ In a meta-analysis comparing metformin and DPP4I, DPP4I was less effective in glycemic control, but it had reduced side effects, such as hypoglycemia and gastrointestinal disturbance.^[18]^

In Korea, RWD studies on the effects of each ADD are limited. An RCT of monotherapy with SU, metformin, and thiazolidinedione in drug-naïve patients with T2D showed comparable glucose-lowering efficacy.^[19]^ Weight gain was more common in the SU and thiazolidinedione groups; hypoglycemia and diarrhea were more common in the SU and metformin groups, respectively.^[19]^

Taken together, the results are inconsistent, and there is a lack of systematic data for comparing the monotherapies used in T2D. Nevertheless, metformin exhibited effective glycemic control and less hypoglycemia, and did not increase the risk of chronic complications. Considering cost-effectiveness, it is reasonable to follow clinical practice guidelines that recommend metformin monotherapy. Other drugs can be considered and chosen according to the degree of hyperglycemia and the risk of hypoglycemia in each patient. SU could be beneficial in patients with significant hyperglycemia and a low risk of hypoglycemia, and DPP4I could be appropriate for patients with moderate hyperglycemia and a relatively high risk of hypoglycemia.

Chronic complications of T2D result from various factors, including hyperglycemia, hypoglycemia, hypertension, and dyslipidemia. In addition to these traditional risk factors, other factors, such as the pleiotropic effects of ADD, can influence the occurrence or progression of chronic complications. Metformin exhibits renoprotective effects in experimental studies and clinical observations.^[20]^ According to a systematic review of 15 eligible studies, metformin was associated with better renal outcomes than SU.^[21]^ DPP4I also exhibits renoprotective effects in T2D.^[22–24]^ In the present study, although statistical significance disappeared after PSM, patients treated with SU were at an increased risk of developing diabetic nephropathy compared with those treated with metformin, and treatment with DPP4I had no significant effect. More patients must be followed up for longer periods to determine the effectiveness of each drug.

Cardiovascular complications of T2D are a major cause of death; therefore, it is important to prevent the development of these complications. The effects of ADD on cardiovascular events and mortality have been extensively evaluated. First-generation SUs have an increased risk of cardiovascular morbidity or mortality hazards.^[17]^ This could be related to hypoglycemia or other mechanisms, such as the effects of myocardial ischemic preconditioning. Second- and third-generation SUs cause less hypoglycemia and comparable cardiovascular effects than OHAs.^[25]^ Furthermore, glycemic variability (GV) and degree of glucose fluctuation are considered important markers of glycemic control in addition to HbA1c levels, which reflect the mean average glycemic status. GV affects the development and progression of various diabetic complications.^[26,27]^ Frequent hypoglycemia and subsequent hyperglycemia due to excessive snacking could be associated with increased GV despite favorable HbA1c levels. Among the 3 OHAs used in our analysis, SU was associated with a significantly higher incidence of hypoglycemia and possibly increased GV, which could ultimately influence the occurrence of chronic complications. In our analysis, the risk of developing IHD was higher in the SU group than in the metformin group before PSM for covariables. However, after PSM, including statin use, statistical significance disappeared. The small sample size may have influenced the results. Analysis of some DPP4I treatment regimens revealed increased HF hospitalizations in cardiovascular outcome trials.^[28]^ However, our analysis did not show a significant difference among the drugs regarding the incidence of newly presenting HF.

In dual combination therapy, metformin + DPP4I therapy was effective in reducing HbA1c levels and reducing the occurrence of hypoglycemia, compared with other dual combinations. This result is unexpected, considering that SU was the most effective monotherapy. In a meta-analysis conducted by Korean researchers, the glucose-lowering efficacy of DPP4I in Asians was superior to that in other ethnicities,^[29]^ which may explain our results. Hypoglycemia is a major barrier to glycemic control; the low risk of hypoglycemia combined with the effects of metformin and DPP4I could also contribute to these results. Another meta-analysis of 10 RCTs revealed comparable efficacy and low hypoglycemia in the SU and DPP4I treatments when combined with metformin.^[30]^ Actually, in clinical practice in Korea, OHA prescription with dual combinations shifted from metformin + SU to metformin + DPP4I, as DPP4I became available.^[5]^ We found no significant differences in the analysis of chronic complications in the dual combination therapy; however, it was difficult to evaluate the effect in the present study because of the small cohort size.

Our study had several strengths. First, we demonstrated the real-world effectiveness and effects on hypoglycemia and chronic complications of 3 frequently prescribed OHAs as monotherapy or dual combination therapies in Korean patients. Few reports have compared the effects of the 3 drugs as monotherapy or dual combination therapies. Second, the present study analyzed OMOP-CDM-transformed EHR data through the open analysis tool provided by OHDSI, which expanded its scope to diabetic research. In a situation where anonymized medical data can be converted into a common structure, and various analysis techniques using big data become possible, this approach could make real-world research easier. Nevertheless, our study had several limitations. First, our CDM data did not include the demographic features of patients, such as weight and body mass index, and clinical information about drug adherence and side effects of OHAs, which are important factors for the interpretation of results. Second, the number of institutions included in the study was small, although this was a multicenter study. Third, the evaluation of chronic complications was based on diagnostic codes or medicines; therefore, there could be cases in which diagnostic codes are omitted or inappropriately added in practice. In addition, in the ATLAS program, patients with pre-existing outcomes were excluded; thus, it was not possible to evaluate progression or recurrence in patients with pre-existing complications. Fourth, as all drugs were classified and analyzed according to the mechanism of action rather than the active ingredient, no differences were identified between drugs. Finally, OHA dose was not considered in the analysis.

In conclusion, this observational study showed that SU followed by metformin was effective as monotherapy, and both drugs showed an increased hazard of reaching HbA1c levels below 7% compared to DPP4I. The dual combination of metformin and DPP4I showed a good glucose-lowering effect and a low risk of hypoglycemia. To decide on OHA prescriptions, clinicians must consider multiple aspects such as glycemic control effects, risk of hypoglycemia, and the impact on chronic complications. Therefore, further studies are warranted to clarify the clinical effectiveness and effects of OHAs (including those of recently introduced OHAs) on various complications of DM in a clinical setting.

## Acknowledgments

We are particularly grateful to Yong-Jin Im and Eun-Young Kim of the Center for Clinical Pharmacology, Biochemical Research Institute in Jeonbuk National University Hospital for their valuable help and support in statistical analyses.

## Author contributions

**Conceptualization:** Tae Sun Park.

**Data curation:** Kyung Ae Lee, Heung Yong Jin.

**Formal analysis:** Kyung Ae Lee, Yu Ji Kim.

**Funding acquisition:** Tae Sun Park.

**Methodology:** Kyung Ae Lee, Heung Yong Jin, Yu Ji Kim, Sang Soo Kim, Eun-Hee Cho.

**Project administration:** Tae Sun Park.

**Visualization:** Kyung Ae Lee.

**Writing – original draft:** Kyung Ae Lee.

**Writing – review & editing:** Kyung Ae Lee, Heung Yong Jin, Tae Sun Park.

## Supplementary Material

Supplemental Digital Content

## Supplementary Material

Supplemental Digital Content

## Supplementary Material

Supplemental Digital Content
