# Supplementary material for: Real-world comparison of mono and dual combination therapies of metformin, sulfonylurea, and dipeptidyl peptidase-4 inhibitors using a common data model: A retrospective observational study
Source: Medicine (Baltimore). 2022 Feb 25;101(8):e28823. doi: 10.1097/MD.0000000000028823 (PMC8878728; doi:10.1097/MD.0000000000028823)
Supplement: Supplemental Digital Content [file medi-101-e28823-s001.docx]

## **Supplementary table 1**

| **Number of patients before and after matching for each drug comparison and outcome hypoglycemia** | | | | | | | | | | | | |
| --- | --- | --- | --- | --- | --- | --- | --- | --- | --- | --- | --- | --- |
| Oral  hypoglycemic agents | JNUH | | | | KNUH | | | | PNUH | | | |
|  | Unmatched | | Matched^a^ | | Unmatched | | Matched^a^ | | Unmatched | | Matched^a^ | |
|  | T | C | T | C | T | C | T | C | T | C | T | C |
| Metformin (T) vs SU (C) | 3,341 | 2,664 | 2,467 | 2,467 | 1,590 | 844 | 844 | 844 | 3,094 | 500 | 500 | 500 |
| Metformin (T) vs DPP4I (C) | 3,341 | 1,213 | 1,191 | 1,191 | 1,590 | 619 | 619 | 619 | 3,094 | 543 | 535 | 535 |
| SU (T) vs DPP4I (C) | 2,664 | 1,213 | 1,136 | 1,136 | 844 | 619 | 577 | 577 | 500 | 543 | 315 | 315 |
| Metformin+DPP4I (T) vs Metformin+SU (C) | 764 | 640 | 597 | 597 | 469 | 274 | 274 | 274 | 964 | 592 | 579 | 579 |
| Metformin+DPP4I (T) vs SU+DPP4I (C) | 764 | 116 | 116 | 116 | 469 | 56 | 56 | 56 | 964 | 99 | 99 | 99 |
| Metformin+SU (T) vs SU+DPP4I (C) | 640 | 116 | 108 | 108 | 274 | 56 | 56 | 56 | 592 | 99 | 97 | 97 |
| SU: Sulfonylureas; DPP4I: DPP-4 inhibitors; T: Target; C: Comparator; | | | | | | | | | | | | |
| ^a^ PSM Covariate: Sex, Age, HbA1c | | | | | | | | | | | | |

| **Number of patients before and after matching for each drug comparison and outcome IHD** | | | | | | | | | | | | |
| --- | --- | --- | --- | --- | --- | --- | --- | --- | --- | --- | --- | --- |
| Oral  hypoglycemic agents | JNUH | | | | KNUH | | | | PNUH | | | |
|  | Unmatched | | Matched^a^ | | Unmatched | | Matched^a^ | | Unmatched | | Matched^a^ | |
|  | T | C | T | C | T | C | T | C | T | C | T | C |
| Metformin (T) vs SU (C) | 1,082 | 823 | 719 | 719 | 548 | 264 | 259 | 259 | 1,065 | 152 | 152 | 152 |
| Metformin (T) vs DPP4I (C) | 1,082 | 390 | 369 | 369 | 548 | 211 | 211 | 211 | 1,065 | 165 | 156 | 156 |
| SU (T) vs DPP4I (C) | 823 | 390 | 310 | 310 | 264 | 211 | 187 | 187 | 422 | 211 | 206 | 206 |
| Metformin+DPP4I (T) vs Metformin+SU (C) | 219 | 161 | 138 | 138 | 178 | 90 | 90 | 90 | 152 | 165 | 80 | 80 |
| Metformin+DPP4I (T) vs SU+DPP4I (C) | 219 | 35 | 35 | 35 | 178 | 16 | 16 | 16 | 422 | 34 | 34 | 34 |
| Metformin+SU (T) vs SU+DPP4I (C) | 161 | 35 | 30 | 30 | 90 | 16 | 16 | 16 | 211 | 34 | 32 | 32 |
| SU: Sulfonylureas; DPP4I: DPP-4 inhibitors; T: Target; C: Comparator; | | | | | | | | | | | | |
| ^a^ PSM Covariate: Sex, Age, HbA1c, Statin | | | | | | | | | | | | |

| **Number of patients before and after matching for each drug comparison and outcome Heart failure** | | | | | | | | | | | | |
| --- | --- | --- | --- | --- | --- | --- | --- | --- | --- | --- | --- | --- |
| Oral  hypoglycemic agents | JNUH | | | | KNUH | | | | PNUH | | | |
|  | Unmatched | | Matched^a^ | | Unmatched | | Matched^a^ | | Unmatched | | Matched^a^ | |
|  | T | C | T | C | T | C | T | C | T | C | T | C |
| Metformin (T) vs SU (C) | 1,325 | 1,044 | 896 | 896 | 560 | 248 | 239 | 239 | 1,362 | 222 | 220 | 220 |
| Metformin (T) vs DPP4I (C) | 1,325 | 506 | 481 | 481 | 560 | 205 | 202 | 202 | 1,362 | 187 | 174 | 174 |
| SU (T) vs DPP4I (C) | 1,044 | 506 | 433 | 433 | 248 | 205 | 180 | 180 | 222 | 187 | 93 | 93 |
| Metformin+DPP4I (T) vs Metformin+SU (C) | 292 | 184 | 164 | 164 | 179 | 71 | 71 | 71 | 448 | 265 | 248 | 248 |
| Metformin+DPP4I (T) vs SU+DPP4I (C) | 292 | 40 | 40 | 40 | 179 | 17 | 17 | 17 | 448 | 35 | 35 | 35 |
| Metformin+SU (T) vs SU+DPP4I (C) | 184 | 40 | 32 | 32 | 71 | 17 | 17 | 17 | 265 | 35 | 33 | 33 |
| SU: Sulfonylureas; DPP4I: DPP-4 inhibitors; T: Target; C: Comparator; | | | | | | | | | | | | |
| ^a^ PSM Covariate: Sex, Age, HbA1c, Statin | | | | | | | | | | | | |

| **Number of patients before and after matching for each drug comparison and outcome Ischemic stroke** | | | | | | | | | | | | |
| --- | --- | --- | --- | --- | --- | --- | --- | --- | --- | --- | --- | --- |
| Oral  hypoglycemic agents | JNUH | | | | KNUH | | | | PNUH | | | |
|  | Unmatched | | Matched^a^ | | Unmatched | | Matched^a^ | | Unmatched | | Matched^a^ | |
|  | T | C | T | C | T | C | T | C | T | C | T | C |
| Metformin (T) vs SU (C) | 1,072 | 846 | 762 | 762 | 572 | 287 | 285 | 285 | 1,410 | 219 | 216 | 216 |
| Metformin (T) vs DPP4I (C) | 1,072 | 445 | 428 | 428 | 572 | 235 | 232 | 232 | 1,410 | 203 | 188 | 188 |
| SU (T) vs DPP4I (C) | 846 | 445 | 392 | 392 | 287 | 235 | 208 | 208 | 219 | 203 | 100 | 100 |
| Metformin+DPP4I (T) vs Metformin+SU (C) | 252 | 166 | 144 | 144 | 190 | 89 | 89 | 89 | 436 | 261 | 243 | 243 |
| Metformin+DPP4I (T) vs SU+DPP4I (C) | 252 | 37 | 37 | 37 | 190 | 16 | 16 | 16 | 436 | 35 | 35 | 35 |
| Metformin+SU (T) vs SU+DPP4I (C) | 166 | 37 | 26 | 26 | 89 | 16 | 16 | 16 | 261 | 35 | 31 | 31 |
| SU: Sulfonylureas; DPP4I: DPP-4 inhibitors; T: Target; C: Comparator; | | | | | | | | | | | | |
| ^a^ PSM Covariate: Sex, Age, HbA1c, Statin | | | | | | | | | | | | |

| **Number of patients before and after matching for each drug comparison and outcome Diabetic retinopathy** | | | | | | | | | | | | |
| --- | --- | --- | --- | --- | --- | --- | --- | --- | --- | --- | --- | --- |
| Oral  hypoglycemic agents | JNUH | | | | KNUH | | | | PNUH | | | |
|  | Unmatched | | Matched^a^ | | Unmatched | | Matched^a^ | | Unmatched | | Matched^a^ | |
|  | T | C | T | C | T | C | T | C | T | C | T | C |
| Metformin (T) vs SU (C) | 1,331 | 1,040 | 897 | 897 | 611 | 293 | 611 | 293 | 1,432 | 230 | 227 | 227 |
| Metformin (T) vs DPP4I (C) | 1,331 | 491 | 474 | 474 | 611 | 222 | 219 | 219 | 1,432 | 189 | 180 | 180 |
| SU (T) vs DPP4I (C) | 1,040 | 491 | 425 | 425 | 293 | 222 | 195 | 195 | 230 | 189 | 100 | 100 |
| Metformin+DPP4I (T) vs Metformin+SU (C) | 290 | 181 | 161 | 161 | 187 | 93 | 93 | 93 | 431 | 263 | 244 | 244 |
| Metformin+DPP4I (T) vs SU+DPP4I (C) | 290 | 39 | 39 | 39 | 187 | 13 | 13 | 13 | 431 | 28 | 28 | 28 |
| Metformin+SU (T) vs SU+DPP4I (C) | 181 | 39 | 32 | 32 | 93 | 13 | 13 | 13 | 263 | 28 | 27 | 27 |
| SU: Sulfonylureas; DPP4I: DPP-4 inhibitors; T: Target; C: Comparator; | | | | | | | | | | | | |
| ^a^ PSM Covariate: Sex, Age, HbA1c, Statin | | | | | | | | | | | | |

| **Number of patients before and after matching for each drug comparison and outcome Diabetic neuropathy** | | | | | | | | | | | | |
| --- | --- | --- | --- | --- | --- | --- | --- | --- | --- | --- | --- | --- |
| Oral  hypoglycemic agents | JNUH | | | | KNUH | | | | PNUH | | | |
|  | Unmatched | | Matched^a^ | | Unmatched | | Matched^a^ | | Unmatched | | Matched^a^ | |
|  | T | C | T | C | T | C | T | C | T | C | T | C |
| Metformin (T) vs SU (C) | 1,142 | 928 | 773 | 773 | 582 | 281 | 277 | 277 | 1,452 | 231 | 227 | 227 |
| Metformin (T) vs DPP4I (C) | 1,142 | 371 | 360 | 360 | 582 | 227 | 220 | 220 | 1,452 | 211 | 202 | 202 |
| SU (T) vs DPP4I (C) | 928 | 371 | 340 | 340 | 281 | 227 | 198 | 198 | 231 | 211 | 105 | 105 |
| Metformin+DPP4I (T) vs Metformin+SU (C) | 240 | 151 | 128 | 128 | 195 | 92 | 92 | 92 | 458 | 264 | 246 | 246 |
| Metformin+DPP4I (T) vs SU+DPP4I (C) | 240 | 32 | 32 | 32 | 195 | 14 | 14 | 14 | 458 | 36 | 36 | 36 |
| Metformin+SU (T) vs SU+DPP4I (C) | 151 | 32 | 23 | 23 | 92 | 14 | 14 | 14 | 264 | 36 | 35 | 35 |
| SU: Sulfonylureas; DPP4I: DPP-4 inhibitors; T: Target; C: Comparator; | | | | | | | | | | | | |
| ^a^ PSM Covariate: Sex, Age, HbA1c, Statin | | | | | | | | | | | | |

| **Number of patients before and after matching for each drug comparison and outcome Diabetic nephropathy** | | | | | | | | | | | | |
| --- | --- | --- | --- | --- | --- | --- | --- | --- | --- | --- | --- | --- |
| Oral  hypoglycemic agents | JNUH | | | | KNUH | | | | PNUH | | | |
|  | Unmatched | | Matched^a^ | | Unmatched | | Matched^a^ | | Unmatched | | Matched^a^ | |
|  | T | C | T | C | T | C | T | C | T | C | T | C |
| Metformin (T) vs SU (C) | 780 | 146 | 146 | 146 | 423 | 142 | 142 | 142 | 1,033 | 140 | 139 | 139 |
| Metformin (T) vs DPP4I (C) | 780 | 219 | 213 | 213 | 423 | 157 | 153 | 153 | 1,033 | 80 | 78 | 78 |
| SU (T) vs DPP4I (C) | 146 | 219 | 138 | 138 | 142 | 157 | 109 | 109 | 140 | 80 | 48 | 48 |
| Metformin+DPP4I (T) vs Metformin+SU (C) | 211 | 56 | 56 | 56 | 153 | 40 | 40 | 40 | 351 | 201 | 191 | 191 |
| Metformin+DPP4I (T) vs SU+DPP4I (C) | 211 | <5 | <5 | <5 | 153 | 10 | 10 | 10 | 351 | 6 | 6 | 6 |
| Metformin+SU (T) vs SU+DPP4I (C) | 56 | <5 | <5 | <5 | NA | NA | NA | NA | 201 | 6 | 6 | 6 |
| SU: Sulfonylureas; DPP4I: DPP-4 inhibitors; T: Target; C: Comparator; | | | | | | | | | | | | |
| ^a^ PSM Covariate: Sex, Age, HbA1c, Statin | | | | | | | | | | | | |

| **Number of patients before and after matching for each drug comparison and outcome UACR** | | | | | | | | | | | | |
| --- | --- | --- | --- | --- | --- | --- | --- | --- | --- | --- | --- | --- |
| Oral  hypoglycemic agents | JNUH | | | | KNUH | | | | PNUH | | | |
|  | Unmatched | | Matched^a^ | | Unmatched | | Matched^a^ | | Unmatched | | Matched^a^ | |
|  | T | C | T | C | T | C | T | C | T | C | T | C |
| Metformin (T) vs SU (C) | 295 | 47 | 47 | 47 | 282 | 87 | 87 | 87 | 476 | 49 | 49 | 49 |
| Metformin (T) vs DPP4I (C) | 295 | 120 | 114 | 114 | 282 | 109 | 109 | 109 | 476 | 48 | 46 | 46 |
| SU (T) vs DPP4I (C) | 47 | 120 | 47 | 47 | 87 | 109 | 68 | 68 | 49 | 48 | 16 | 16 |
| Metformin+DPP4I (T) vs Metformin+SU (C) | 92 | 28 | 28 | 28 | 78 | 29 | 29 | 29 | 263 | 98 | 98 | 98 |
| Metformin+DPP4I (T) vs SU+DPP4I (C) | 92 | <5 | <5 | <5 | 78 | 10 | 10 | 10 | 263 | 6 | 6 | 6 |
| Metformin+SU (T) vs SU+DPP4I (C) | NA | NA | NA | NA | NA | NA | NA | NA | 98 | 6 | 5 | 5 |
| SU: Sulfonylureas; DPP4I: DPP-4 inhibitors; T: Target; C: Comparator; | | | | | | | | | | | | |
| ^a^ PSM Covariate: Sex, Age, HbA1c, Statin | | | | | | | | | | | | |
