# Supplementary material for: Real-world comparison of mono and dual combination therapies of metformin, sulfonylurea, and dipeptidyl peptidase-4 inhibitors using a common data model: A retrospective observational study
Source: Medicine (Baltimore). 2022 Feb 25;101(8):e28823. doi: 10.1097/MD.0000000000028823 (PMC8878728; doi:10.1097/MD.0000000000028823)
Supplement: Supplemental Digital Content [file medi-101-e28823-s002.docx]

## **Supplementary table 2**

| **Number of Patients, Hazard Ratio, Confidence Intervals (CI), *P* Values and Calibrated *P* Values for Each Drug Comparison and Each Outcome Based on Analysis Across All Three Study Sites.** | | | | | | |
| --- | --- | --- | --- | --- | --- | --- |
| Study Site | N | Hazard Ratio | 95% CI Lower bound | 95% CI Upper bound | *P*-Value | Calibrated *P*-Value |
| Comparison: Metformin vs SU – Outcome: HbA1c^a^ | | | | | | |
| JNUH | 1,147 | 0.77 | 0.65 | 0.92 | 0.00 | NA |
| KNUH | 556 | 1.02 | 0.80 | 1.29 | 0.90 | 0.23 |
| PNUH | 243 | 0.91 | 0.64 | 1.29 | 0.60 | NA |
| Comparison: Metformin vs SU – Outcome: Hypoglycemia^b^ | | | | | | |
| JNUH | 2,467 | 0.31 | 0.20 | 0.45 | 0.00 | NA |
| KNUH | 844 | 0.29 | 0.10 | 0.74 | 0.02 | 0.00 |
| PNUH | 500 | 0.14 | 0.01 | 0.80 | 0.10 | 0.23 |
| Comparison: Metformin vs SU – Outcome: IHD^c^ | | | | | | |
| JNUH | 719 | 0.31 | 0.15 | 0.58 | 0.00 | NA |
| KNUH | 259 | 1.67 | 0.74 | 3.97 | 0.23 | 0.70 |
| PNUH | 152 | 0.57 | 0.23 | 1.33 | 0.21 | NA |
| Comparison: Metformin vs SU – Outcome: Heart failure^c^ | | | | | | |
| JNUH | 896 | 0.13 | 0.02 | 0.47 | 0.01 | NA |
| KNUH | 239 | 0.79 | 0.39 | 1.55 | 0.50 | 0.14 |
| PNUH | 220 | 0.64 | 0.23 | 1.62 | 0.36 | NA |
| Comparison: Metformin vs SU – Outcome: Ischemic stroke^c^ | | | | | | |
| JNUH | 762 | 0.54 | 0.27 | 1.02 | 0.06 | NA |
| KNUH | 285 | 1.40 | 0.45 | 4.73 | 0.58 | 0.79 |
| PNUH | 216 | 0.86 | 0.28 | 2.58 | 0.79 | NA |
| Comparison: Metformin vs SU – Outcome: Diabetic retinopathy^c^ | | | | | | |
| JNUH | 897 | 0.87 | 0.48 | 1.57 | 0.66 | NA |
| KNUH | 287 | 1.59 | 1.03 | 2.50 | 0.04 | 0.58 |
| PNUH | 227 | 1.50 | 0.54 | 4.47 | 0.45 | NA |
| Comparison: Metformin vs SU – Outcome: Diabetic neuropathy^c^ | | | | | | |
| JNUH | 773 | 0.80 | 0.59 | 1.10 | 0.18 | NA |
| KNUH | 277 | 0.73 | 0.38 | 1.38 | 0.34 | 0.08 |
| PNUH | 227 | 0.80 | 0.20 | 3.02 | 0.75 | NA |
| Comparison: Metformin vs SU – Outcome: Diabetic nephropathy^c^ | | | | | | |
| JNUH | 146 | 1.27 | 0.65 | 2.53 | 0.50 | NA |
| KNUH | 142 | 0.44 | 0.23 | 0.79 | 0.01 | 0.00 |
| PNUH | 139 | 0.74 | 0.43 | 1.27 | 0.28 | NA |
| Comparison: Metformin vs SU – Outcome: UACR^c^ | | | | | | |
| JNUH | 47 | 2.50 | 0.84 | 9.11 | 0.13 | NA |
| KNUH | 87 | 0.33 | 0.14 | 0.71 | 0.01 | NA |
| PNUH | 49 | 0.50 | 0.20 | 1.14 | 0.12 | NA |
| Comparison: Metformin vs DPP4I – Outcome: HbA1c^a^ | | | | | | |
| JNUH | 721 | 2.05 | 1.57 | 2.70 | 0.00 | NA |
| KNUH | 484 | 1.66 | 1.25 | 2.21 | 0.00 | 0.00 |
| PNUH | 286 | 1.24 | 0.88 | 1.76 | 0.22 | 0.25 |
| Comparison: Metformin vs DPP4I – Outcome: Hypoglycemia^b^ | | | | | | |
| JNUH | 1,191 | 0.85 | 0.44 | 1.62 | 0.63 | NA |
| KNUH | 619 | 2.67 | 0.77 | 12.18 | 0.16 | 0.05 |
| PNUH | 535 | 1.00 | 0.12 | 8.33 | 1.00 | 0.76 |
| Comparison: Metformin vs DPP4I – Outcome: IHD^c^ | | | | | | |
| JNUH | 369 | 0.41 | 0.16 | 0.95 | 0.05 | NA |
| KNUH | 211 | 1.09 | 0.48 | 2.51 | 0.84 | 0.69 |
| PNUH | 156 | 1.50 | 0.43 | 5.87 | 0.54 | NA |
| Comparison: Metformin vs DPP4I – Outcome: Heart failure^c^ | | | | | | |
| JNUH | 481 | 0.25 | 0.04 | 1.00 | 0.10 | NA |
| KNUH | 202 | 0.81 | 0.38 | 1.69 | 0.58 | 0.65 |
| PNUH | 174 | 3.50 | 0.85 | 23.49 | 0.14 | NA |
| Comparison: Metformin vs DPP4I – Outcome: Ischemic stroke^c^ | | | | | | |
| JNUH | 428 | 1.57 | 0.62 | 4.27 | 0.36 | NA |
| KNUH | 232 | 0.29 | 0.04 | 1.18 | 0.14 | 0.17 |
| PNUH | 188 | 2.00 | 0.39 | 14.42 | 0.45 | NA |
| Comparison: Metformin vs DPP4I – Outcome: Diabetic retinopathy^c^ | | | | | | |
| JNUH | 474 | 0.71 | 0.31 | 1.60 | 0.42 | NA |
| KNUH | 219 | 0.93 | 0.55 | 1.57 | 0.79 | 0.64 |
| PNUH | 180 | 0.75 | 0.25 | 2.16 | 0.60 | NA |
| Comparison: Metformin vs DPP4I – Outcome: Diabetic neuropathy^c^ | | | | | | |
| JNUH | 360 | 1.23 | 0.73 | 2.08 | 0.43 | NA |
| KNUH | 220 | 1.75 | 0.75 | 4.38 | 0.21 | 0.24 |
| PNUH | 202 | 0.67 | 0.22 | 1.85 | 0.45 | NA |
| Comparison: Metformin vs DPP4I – Outcome: Diabetic nephropathy^c^ | | | | | | |
| JNUH | 213 | 1.14 | 0.64 | 2.07 | 0.66 | NA |
| KNUH | 153 | 0.73 | 0.40 | 1.31 | 0.30 | 0.56 |
| PNUH | 78 | 0.38 | 0.12 | 1.02 | 0.08 | 0.42 |
| Comparison: Metformin vs DPP4I – Outcome: UACR^c^ | | | | | | |
| JNUH | 114 | 2.00 | 0.99 | 4.28 | 0.06 | NA |
| KNUH | 109 | 0.75 | 0.38 | 1.46 | 0.40 | 0.48 |
| PNUH | 46 | 0.40 | 0.11 | 1.20 | 0.13 | NA |
| Comparison: SU vs DPP4I – Outcome: HbA1c^a^ | | | | | | |
| JNUH | 702 | 2.43 | 1.88 | 3.17 | 0.00 | NA |
| KNUH | 461 | 1.89 | 1.40 | 2.58 | 0.00 | 0.00 |
| PNUH | 226 | 1.47 | 1.01 | 2.15 | 0.05 | 0.00 |
| Comparison: SU vs DPP4I – Outcome: Hypoglycemia^b^ | | | | | | |
| JNUH | 1,136 | 4.42 | 2.45 | 8.67 | 0.00 | NA |
| KNUH | 577 | 16.00 | 3.27 | NA | 0.02 | 0.01 |
| PNUH | 315 | NA | NA | NA | NA | NA |
| Comparison: SU vs DPP4I – Outcome: IHD^c^ | | | | | | |
| JNUH | 310 | 2.25 | 1.01 | 5.48 | 0.06 | NA |
| KNUH | 187 | 0.46 | 0.16 | 1.17 | 0.12 | 0.53 |
| PNUH | 80 | 2.33 | 0.65 | 10.83 | 0.24 | NA |
| Comparison: SU vs DPP4I – Outcome: Heart failure^c^ | | | | | | |
| JNUH | 433 | 1.29 | 0.48 | 3.60 | 0.63 | NA |
| KNUH | 180 | 0.92 | 0.42 | 2.04 | 0.84 | 0.44 |
| PNUH | 93 | 1.50 | 0.25 | 11.39 | 0.68 | NA |
| Comparison: SU vs DPP4I – Outcome: Ischemic stroke^c^ | | | | | | |
| JNUH | 392 | 3.33 | 1.42 | 9.11 | 0.01 | NA |
| KNUH | 208 | 0.33 | 0.05 | 1.45 | 0.20 | 0.46 |
| PNUH | 100 | 1.00 | 0.04 | 25.27 | 1.00 | NA |
| Comparison: SU vs DPP4I – Outcome: Diabetic retinopathy^c^ | | | | | | |
| JNUH | 425 | 0.50 | 0.17 | 1.29 | 0.17 | NA |
| KNUH | 195 | 1.32 | 0.73 | 2.42 | 0.37 | 0.05 |
| PNUH | 100 | 0.50 | 0.11 | 1.90 | 0.35 | NA |
| Comparison: SU vs DPP4I – Outcome: Diabetic neuropathy^c^ | | | | | | |
| JNUH | 340 | 1.25 | 0.76 | 2.07 | 0.38 | NA |
| KNUH | 198 | 1.78 | 0.80 | 4.20 | 0.17 | 0.03 |
| PNUH | 105 | 0.33 | 0.05 | 1.45 | 0.20 | NA |
| Comparison: SU vs DPP4I – Outcome: Diabetic nephropathy^c^ | | | | | | |
| JNUH | 138 | 1.14 | 0.56 | 2.37 | 0.72 | NA |
| KNUH | 109 | 1.24 | 0.65 | 2.37 | 0.52 | 0.08 |
| PNUH | 48 | 0.78 | 0.28 | 2.09 | 0.63 | NA |
| Comparison: SU vs DPP4I – Outcome: UACR^c^ | | | | | | |
| JNUH | 47 | 1.50 | 0.43 | 5.87 | 0.54 | NA |
| KNUH | 68 | 0.87 | 0.42 | 1.80 | 0.72 | 0.51 |
| PNUH | 16 | 0.80 | 0.20 | 3.02 | 0.75 | NA |
| Comparison: Metformin+DPP4I vs Metformin+SU – Outcome: HbA1c^a^ | | | | | | |
| JNUH | 241 | 1.16 | 0.83 | 1.63 | 0.39 | NA |
| KNUH | 169 | 1.04 | 0.70 | 1.56 | 0.84 | NA |
| PNUH | 328 | 1.28 | 0.99 | 1.65 | 0.06 | 0.19 |
| Comparison: Metformin+DPP4I vs Metformin+SU – Outcome: Hypoglycemia^b^ | | | | | | |
| JNUH | 597 | 0.12 | 0.03 | 0.33 | 0.00 | NA |
| KNUH | 274 | 0.12 | 0.01 | 0.68 | 0.08 | NA |
| PNUH | 579 | 0.14 | 0.02 | 0.51 | 0.01 | 0.11 |
| Comparison: Metformin+DPP4I vs Metformin+SU – Outcome: IHD^c^ | | | | | | |
| JNUH | 138 | 1.50 | 0.43 | 5.87 | 0.54 | NA |
| KNUH | 90 | 0.29 | 0.04 | 1.18 | 0.14 | NA |
| PNUH | 206 | 1.00 | 0.34 | 2.92 | 1.00 | 0.50 |
| Comparison: Metformin+DPP4I vs Metformin+SU – Outcome: Heart failure^c^ | | | | | | |
| JNUH | 164 | 0.00 | NA | 1.61 | NA | NA |
| KNUH | 71 | 1.83 | 0.70 | 5.32 | 0.24 | NA |
| PNUH | 248 | 0.42 | 0.17 | 0.93 | 0.04 | 0.01 |
| Comparison: Metformin+DPP4I vs Metformin+SU – Outcome: Ischemic stroke^c^ | | | | | | |
| JNUH | 144 | 3.00 | 0.38 | 60.62 | 0.39 | NA |
| KNUH | 89 | 0.60 | 0.12 | 2.45 | 0.50 | NA |
| PNUH | 243 | 0.38 | 0.08 | 1.30 | 0.16 | 0.07 |
| Comparison: Metformin+DPP4I vs Metformin+SU – Outcome: Diabetic retinopathy^c^ | | | | | | |
| JNUH | 161 | 1.00 | 0.39 | 2.56 | 1.00 | NA |
| KNUH | 93 | 1.42 | 0.68 | 3.04 | 0.36 | NA |
| PNUH | 244 | 1.92 | 1.00 | 3.88 | 0.06 | 0.55 |
| Comparison: Metformin+DPP4I vs Metformin+SU – Outcome: Diabetic neuropathy^c^ | | | | | | |
| JNUH | 128 | 0.43 | 0.19 | 0.91 | 0.04 | NA |
| KNUH | 92 | 0.35 | 0.13 | 0.85 | 0.03 | NA |
| PNUH | 246 | 3.00 | 0.90 | 13.52 | 0.11 | 0.40 |
| Comparison: Metformin+DPP4I vs Metformin+SU – Outcome: Diabetic nephropathy^c^ | | | | | | |
| JNUH | 56 | 0.44 | 0.12 | 1.36 | 0.19 | NA |
| KNUH | 40 | 0.90 | 0.36 | 2.23 | 0.82 | NA |
| PNUH | 191 | 0.93 | 0.61 | 1.42 | 0.75 | NA |
| Comparison: Metformin+DPP4I vs Metformin+SU – Outcome: UACR^c^ | | | | | | |
| JNUH | 28 | 0.50 | 0.11 | 1.90 | 0.35 | NA |
| KNUH | 29 | 1.17 | 0.39 | 3.62 | 0.79 | NA |
| PNUH | 98 | 0.87 | 0.47 | 1.58 | 0.65 | NA |
| Comparison: Metformin+DPP4I vs SU+DPP4I - Outcome: HbA1c^a^ | | | | | | |
| JNUH | 60 | 3.40 | 1.34 | 10.34 | 0.02 | NA |
| KNUH | 35 | 1.08 | 0.49 | 2.41 | 0.84 | NA |
| PNUH | 49 | 2.36 | 1.20 | 4.99 | 0.02 | NA |
| Comparison: Metformin+DPP4I vs SU+DPP4I - Outcome: Hypoglycemia^b^ | | | | | | |
| JNUH | 116 | 0.33 | 0.02 | 2.60 | 0.39 | NA |
| KNUH | 56 | 0.14 | NA | 6.75 | NA | NA |
| PNUH | 99 | 0.14 | NA | 1.15 | NA | NA |
| Comparison: Metformin+DPP4I vs SU+DPP4I – Outcome: IHD^c^ | | | | | | |
| JNUH | 35 | 1.00 | 0.12 | 8.33 | 1.00 | NA |
| KNUH | 16 | NA | NA | NA | NA | NA |
| PNUH | 34 | 1.00 | 0.04 | 25.27 | 1.00 | NA |
| Comparison: Metformin+DPP4I vs SU+DPP4I – Outcome: Heart failure^c^ | | | | | | |
| JNUH | 40 | NA | NA | NA | NA | NA |
| KNUH | 17 | 0.00 | 0.00 | 5.83 | 0.36 | NA |
| PNUH | 35 | 0.33 | 0.02 | 2.60 | 0.39 | NA |
| Comparison: Metformin+DPP4I vs SU+DPP4I – Outcome: Ischemic stroke^c^ | | | | | | |
| JNUH | 37 | 2.00 | 0.19 | 43.01 | 0.62 | NA |
| KNUH | 16 | NA | NA | NA | NA | NA |
| PNUH | 35 | NA | NA | NA | NA | NA |
| Comparison: Metformin+DPP4I vs SU+DPP4I – Outcome: Diabetic retinopathy^c^ | | | | | | |
| JNUH | 39 | 0.50 | 0.07 | 2.56 | 0.45 | NA |
| KNUH | 13 | 1.00 | 0.04 | 25.27 | 1.00 | NA |
| PNUH | 28 | 0.50 | 0.07 | 2.56 | 0.45 | NA |
| Comparison: Metformin+DPP4I vs SU+DPP4I – Outcome: Diabetic neuropathy^c^ | | | | | | |
| JNUH | 32 | 0.33 | 0.05 | 1.45 | 0.20 | NA |
| KNUH | 14 | NA | NA | NA | NA | NA |
| PNUH | 36 | 0.33 | 0.02 | 2.60 | 0.39 | NA |
| Comparison: Metformin+DPP4I vs SU+DPP4I – Outcome: Diabetic nephropathy^c^ | | | | | | |
| JNUH | <5 | NA | NA | NA | NA | NA |
| KNUH | 10 | 0.20 | 0.01 | 1.24 | 0.19 | NA |
| PNUH | 6 | 0.50 | 0.02 | 5.22 | 0.62 | NA |
| Comparison: Metformin+DPP4I vs SU+DPP4I – Outcome: UACR^c^ | | | | | | |
| JNUH | <5 | NA | NA | NA | NA | NA |
| KNUH | 10 | 0.20 | 0.01 | 1.24 | 0.19 | NA |
| PNUH | 6 | 0.14 | NA | 0.84 | NA | NA |
| Comparison: Metformin+SU vs SU+DPP4I - Outcome: HbA1c^a^ | | | | | | |
| JNUH | 56 | 2.57 | 1.12 | 6.61 | 0.04 | NA |
| KNUH | 35 | 0.87 | 0.31 | 2.44 | 0.80 | NA |
| PNUH | 49 | 1.69 | 0.86 | 3.45 | 0.14 | NA |
| Comparison: Metformin+SU vs SU+DPP4I – Outcome: Hypoglycemia^b^ | | | | | | |
| JNUH | 108 | 2.00 | 0.63 | 7.49 | 0.27 | NA |
| KNUH | 56 | NA | NA | NA | NA | NA |
| PNUH | 97 | 0.00 | NA | 1.61 | NA | NA |
| Comparison: Metformin+SU vs SU+DPP4I – Outcome: IHD^c^ | | | | | | |
| JNUH | 30 | 0.14 | NA | 1.97 | NA | NA |
| KNUH | 16 | NA | NA | NA | NA | NA |
| PNUH | 32 | 3.00 | 0.38 | 60.62 | 0.39 | NA |
| Comparison: Metformin+SU vs SU+DPP4I – Outcome: Heart failure^c^ | | | | | | |
| JNUH | 32 | NA | NA | NA | NA | NA |
| KNUH | 17 | 0.14 | NA | 6.75 | NA | NA |
| PNUH | 33 | 1.00 | 0.19 | 5.40 | 1.00 | NA |
| Comparison: Metformin+SU vs SU+DPP4I – Outcome: Ischemic stroke^c^ | | | | | | |
| JNUH | 26 | NA | NA | NA | NA | NA |
| KNUH | 16 | NA | NA | NA | NA | NA |
| PNUH | 31 | NA | NA | NA | NA | NA |
| Comparison: Metformin+SU vs SU+DPP4I – Outcome: Diabetic retinopathy^c^ | | | | | | |
| JNUH | 32 | 0.25 | 0.01 | 1.69 | 0.27 | NA |
| KNUH | 13 | 4.00 | 0.59 | 78.20 | 0.27 | NA |
| PNUH | 27 | 0.00 | NA | 0.62 | NA | NA |
| Comparison: Metformin+SU vs SU+DPP4I – Outcome: Diabetic neuropathy^c^ | | | | | | |
| JNUH | 5 | 0.00 | 0.00 | 5.83 | 0.22 | NA |
| KNUH | 14 | NA | NA | NA | NA | NA |
| PNUH | 35 | 0.14 | NA | 1.15 | NA | NA |
| Comparison: Metformin+SU vs SU+DPP4I – Outcome: Diabetic nephropathy^c^ | | | | | | |
| JNUH | NA | NA | NA | NA | NA | NA |
| KNUH | NA | NA | NA | NA | NA | NA |
| PNUH | 6 | 0.50 | 0.02 | 5.22 | 0.62 | NA |
| Comparison: Metformin+SU vs SU+DPP4I – Outcome: UACR^c^ | | | | | | |
| JNUH | NA | NA | NA | NA | NA | NA |
| KNUH | NA | NA | NA | NA | NA | NA |
| PNUH | 5 | 0.14 | NA | 1.97 | NA | NA |
| SU: Sulfonylureas; DPP4I: DPP-4 inhibitors; N: No. of patients; CI: Confidence interval | | | | | | |
| ^a^ PSM Covariate: Sex, Age | | | | | | |
| ^b^ PSM Covariate: Sex, Age, HbA1c | | | | | | |
| ^c^ PSM Covariate: Sex, Age, HbA1c, Statin | | | | | | |
