# Supplementary material for: Real-world comparison of mono and dual combination therapies of metformin, sulfonylurea, and dipeptidyl peptidase-4 inhibitors using a common data model: A retrospective observational study
Source: Medicine (Baltimore). 2022 Feb 25;101(8):e28823. doi: 10.1097/MD.0000000000028823 (PMC8878728; doi:10.1097/MD.0000000000028823)
Supplement: Supplemental Digital Content [file medi-101-e28823-s003.docx]

## **Supplementary table 3**

| **Hazard ratios (HRs) for hypoglycemia and chronic complications of dual theraphy (after meta-analysis)** | | | |
| --- | --- | --- | --- |
| Outcome | Consensus Hazard Ratio (95% CI) | | |
|  | Metformin+DPP4I (T) vs Metformin+SU (C) | Metformin+DPP4I (T) vs SU+DPP4I (C) | Metformin+SU (T) vs SU+DPP4I (C) |
| Hypoglycemia^a^ | 0.16 (0.07 – 0.36) | 0.33 (0.03 – 3.76)^d^ | 1.50 (0.48 – 4.64)^i^ |
| Hypoglycemia^b^ | 0.13 (0.05 – 0.30) | 0.33 (0.03 – 3.76)^d^ | 2.00 (0.58 – 6.90)^d^ |
| IHD^a^ | 0.82 (0.36 – 1.84) | 1.50 (0.22 – 10.12)^d^ | 1.00 (0.17 – 5.88)^i^ |
| IHD^b^ | 0.87 (0.40 – 1.89) | 1.50 (0.19 – 11.95)^i^ | 1.00 (0.04 – 25.13)^f^ |
| IHD^c^ | 0.90 (0.43 – 1.89) | 1.00 (0.17 – 5.88)^i^ | 3.00 (0.24 – 37.89)^f^ |
| Heart failure^a^ | 0.60 (0.30 – 1.17) | 1.00 (0.19 – 5.33)^f^ | 1.00 (0.19 – 5.33)^f^ |
| Heart failure^b^ | 0.89 (0.26 – 3.12) | 0.33 (0.03 – 3.76)^f^ | 0.67 (0.1 – 4.48)^f^ |
| Heart failure^c^ | 0.85 (0.20 – 3.61)^h^ | 0.33 (0.03 – 3.76)^f^ | 1.00 (0.19 – 5.33)^f^ |
| Ischemic stroke^a^ | 0.60 (0.24 – 1.54)^h^ | 1.00 (0.04 – 25.13)^d^ | NA |
| Ischemic stroke^b^ | 0.54 (0.21 – 1.41)^h^ | NA | 1.00 (0.04 – 25.13)^d^ |
| Ischemic stroke^c^ | 0.61 (0.24 – 1.57) | 2.00 (0.13 – 30.09)^d^ | NA |
| Diabetic retinopathy^a^ | 1.33 (0.86 – 2.04) | 0.59 (0.12 – 2.84)^g^ | 0.86 (0.24 – 3.14) |
| Diabetic retinopathy^b^ | 1.45 (0.91 – 2.30) | 0.77 (0.27 – 2.20) | 0.91 (0.21 – 3.94) |
| Diabetic retinopathy^c^ | 1.49 (0.96 – 2.33) | 0.55 (0.17 – 1.79) | 1.07 (0.18 – 6.27)^g^ |
| Diabetic neuropathy^a^ | 0.61 (0.16 – 2.28) | 0.42 (0.09 – 1.87)^i^ | 0.50 (0.03 – 8.08)^f^ |
| Diabetic neuropathy^b^ | 0.57 (0.14 – 2.28) | 0.23 (0.04 – 1.23)^i^ | 1.00 (0.04 – 25.13)^d^ |
| Diabetic neuropathy^c^ | 0.68 (0.23 – 2.05) | 0.33 (0.08 – 1.32)^e^ | NA |
| Diabetic nephropathy^a^ | 0.65 (0.45 – 0.93) | 0.20 (0.02 – 2.23)^e^ | NA |
| Diabetic nephropathy^b^ | 0.74 (0.52 – 1.07) | 0.20 (0.02 – 2.23)^e^ | NA |
| Diabetic nephropathy^c^ | 0.86 (0.60 – 1.25) | 0.30 (0.05 – 1.83)^h^ | 0.50 (0.03 – 8.08)^f^ |
| UACR ≥ 30^a^ | 0.88 (0.53 – 1.48) | 0.20 (0.02 – 2.23)^e^ | 0.33 (0.03 – 3.76)^f^ |
| UACR ≥ 30^b^ | 0.75 (0.45 – 1.26) | 0.20 (0.02 – 2.23)^e^ | NA |
| UACR ≥ 30^c^ | 0.86 (0.52 – 1.42) | 0.20 (0.02 – 2.23)^e^ | NA |
| SU: Sulfonylureas; DPP4I: dipeptidyl peptidase -4 inhibitors; T: Target; C: Comparator; CI: Confidence interval; | | | |
| ^a^ PSM Covariate: Sex, Age | | | |
| ^b^ PSM Covariate: Sex, Age, HbA1c | | | |
| ^c^ PSM Covariate: Sex, Age, HbA1c, Statin | | | |
| ^d^ Analysis result of JNUH because data from KNUH and PNUH were unavailable | | | |
| ^e^ Analysis result of KNUH because data from JNUH and PNUH were unavailable | | | |
| ^f^ Analysis result of PNUH because data from JNUH and KNUH were unavailable | | | |
| ^g^ Meta-analysis result of JNUH and KNUH because data from PNUH was unavailable | | | |
| ^h^ Meta-analysis result of KNUH and PNUH because data from JNUH was unavailable | | | |
| ^i^ Meta-analysis result of JNUH and PNUH because data from KNUH was unavailable | | | |
